# MicroRNAs Encoded by Bovine Leukemia Virus (BLV) Are Associated with Reduced Expression of B Cell Transcriptional Regulators in Dairy Cattle Naturally Infected with BLV

**DOI:** 10.3389/fvets.2017.00245

**Published:** 2018-01-15

**Authors:** Meredith C. Frie, Casey J. Droscha, Ashley E. Greenlick, Paul M. Coussens

**Affiliations:** ^1^Cell and Molecular Biology Program, Michigan State University, East Lansing, MI, United States; ^2^NorthStar Cooperative, East Lansing, MI, United States; ^3^Department of Animal Science, Michigan State University, East Lansing, MI, United States

**Keywords:** bovine leukemia virus, microRNA, antibody, IgM, *TAX*, BLIMP1, BCL6, IGJ

## Abstract

Bovine leukemia virus (BLV) is estimated to infect over 83% of dairy herds and over 40% of all dairy cows in the United States. While, BLV only causes leukemia in a small proportion of animals, research indicates that BLV+ cattle exhibit reduced milk production and longevity that is distinct from lymphoma development. It is hypothesized that BLV negatively affects production by interfering with cattle immunity and increasing the risk of secondary infections. In particular, BLV+ cows demonstrate reduced circulating levels of both antigen-specific and total IgM. This study investigated possible mechanisms by which BLV could interfere with the production of IgM in naturally infected cattle. Specifically, total plasma IgM and the expression of genes *IGJ, BLIMP1, BCL6*, and *PAX5* in circulating IgM+ B cells were measured in 15 naturally infected BLV+ and 15 BLV− cows. In addition, BLV proviral load (PVL) (a relative measurement of BLV provirus integrated into host DNA) and the relative expression of BLV *TAX* and 5 BLV microRNAs (miRNAs) were characterized and correlated to the expression of selected endogenous genes. BLV+ cows exhibited lower total plasma IgM and lower expression of *IGJ, BLIMP1*, and *BCL6*. While, BLV *TAX* and BLV miRNAs failed to correlate with *IGJ* expression, both BLV *TAX* and BLV miRNAs exhibited negative associations with *BLIMP1* and *BCL6* gene expression. The results suggest a possible transcriptional pathway by which BLV interferes with IgM production in naturally infected cattle.

## Introduction

Bovine leukemia virus (BLV) is a δ-retrovirus that is the causative agent of enzootic bovine leukosis (EBL) (1). While the clinical stage of EBL is typically characterized by lymphosarcoma development, only a small percentage of infected cattle will progress to clinical disease (2). As a result, dairy producers rarely test for BLV infection on commercial dairy operations. However, it is estimated that at least 83% of US dairy herds are BLV-infected. The within-herd infection rate is often between 25 and nearly 50%, and approximately 40% of all dairy cows in the US are infected (3).

While lymphosarcoma development is rare, approximately 30% of infected cattle will develop persistent lymphocytosis (PL), a benign, polyclonal expansion of the B-cell compartment (2) that is associated with an increased proviral load (PVL) (4). In addition, a growing body of research strongly suggests that BLV negatively interferes with immune function in infected cattle (5). One striking phenotype is that BLV-infected cattle exhibit abnormal levels of serum IgM. BLV+ cattle exhibit lower total IgM (6, 7); lower antigen-specific IgM (8–10); and lower transcript levels encoding the immunoglobulin light chain in IgM+ B cells (11). What is puzzling is a potential mechanism by which BLV interferes with immune function. Although BLV is primarily transmitted within a host by cell-to-cell contact during early infection (12), chronic viral transmission within a host is characterized by proviral replication *via* the division of infected cells. However, multiple studies suggest that BLV is mostly transcriptionally inactive when attempting to detect transcripts of BLV protein-coding genes. Indeed, detecting BLV proteins is nearly impossible *ex vivo* (13), and evidence suggests that BLV protein expression is associated with rapid clearance of infected cells (14).

Recently, researchers discovered that the BLV genome encodes 5 pre-microRNA (miRNA) hairpins that produce 10 mature miRNAs in a genomic region previously thought to be inactive (15, 16). BLV miRNAs are atypical in several ways; virally encoded miRNAs are unusual in RNA viruses due to the potential for cleaving genomic RNA (15) and BLV miRNAs are transcribed by RNA polymerase III (RNAPIII), as opposed to the typical miRNA biosynthesis that utilizes RNAPII (15–18). Despite these unusual features, BLV miRNAs have been detected at levels equivalent to endogenous miRNAs in both bovine B-cell tumors (16) and in experimentally infected bovine calves (19).

Bovine leukemia virus miRNAs also provide a possible mechanism by which “silent” proviruses interfere with host immunity, because evidence suggests that transcription of BLV miRNAs by RNAPIII is independent of the transcription of BLV protein-coding genes by RNAPII (16). Research investigating the role of BLV miRNAs in BLV pathogenesis identified multiple potential genes that could be targeted by BLV miRNAs, including *IGJ* (19). *IGJ* encodes the J chain, which is essential for the assembly and secretion of pentameric IgM and dimeric IgA, as well as for the transport of polymeric IgM and IgA across epithelial barriers *via* the polymeric immunoglobulin receptor (20). In addition, *IGJ* expression has been found to be downregulated in peripheral blood isolated from BLV+ cows (21).

*IGJ* transcription occurs in terminally differentiating B cells that will become plasma cells, which secrete high levels of antibodies (22). B-cell terminal differentiation is tightly regulated by a complex program of transcription factors. PAX5 is considered the master regulator of the B-cell lineage and is expressed consistently from pro-B cells to mature B cells and its expression only declines after terminal differentiation (23). BCL6 is a negative transcriptional regulator that specifically targets genes associated with DNA damage sensing, DNA damage response, and DNA damage checkpoints. This is essential because BCL6 is primarily active in B cells during immunoglobulin light chain rearrangement and in germinal center reactions, during which somatic hypermutation and affinity maturation occur (24). Indeed, mice without *BCL6* expression fail to develop germinal centers and produce low-affinity IgG (25). Of note, *BCL6* upregulation is also associated with the development of B-cell lymphomas in humans (26). Finally, *BLIMP1* is considered the master regulator of plasma cell differentiation and will induce *IGJ* transcription (23). While, *PAX5* and *BCL6* repress *BLIMP1* expression, *BLIMP1*, once expressed, will in turn repress *PAX5* and *BCL6* expression (23).

In this study, we investigated whether BLV+ cows exhibited lower total IgM, as well as lower expression of genes associated with B-cell function and differentiation, specifically *IGJ, PAX5, BLIMP1*, and *BCL6*. In addition, we investigated whether BLV PVL, BLV *TAX* expression, or BLV miRNA expression correlated to measured phenotypes in BLV+ cows. We observed that BLV+ cows exhibited lower total plasma IgM, as well as lower *IGJ, BLIMP1*, and *BCL6* expression. In addition, we were able to detect both BLV *TAX* expression and BLV miRNA expression. Finally, both BLV *TAX* and BLV miRNA expression correlated with reduced B-cell function or gene expression. These data suggest that lower plasma IgM in BLV+ cows could be related to viral interference in the transcriptional regulation that controls plasma cell differentiation.

## Materials and Methods

### Animals

About 30 adults, lactating Holstein cows housed at a commercial dairy farm in mid-Michigan were enrolled in this study. BLV+ cattles were initially selected on the basis of a positive milk using ELISA diagnostic test (NorthStar Cooperative, Inc.,), and were subsequently screened based on their total leukocyte counts (TLC). BLV+ status was confirmed using a serum ELISA diagnostic test (NorthStar Cooperative, Inc.,). BLV+ cows were then matched with BLV− control cows (BLV status as determined by prior milk ELISA diagnostic test and confirmed using a serum ELISA diagnostic test) based upon age, lactation number, days in milk, reproduction status, and days until parturition (Table 1). All animal use protocols were reviewed and approved by Michigan State University Institutional Animal Use and Care Committee (AUF# 04/16-061-00), and written permission was obtained from the commercial dairy herd owner.

**Table 1:** Cow enrollment characteristics.

	BLV+ cows (*n* = 15)	BLV− cows (*n* = 15)
Age	4 years 8 months (3 years 3 months–8 years 1 month)	4 years 8 months (2 years 11 months–8 years 1 month)
Lactation number	2.7 (1–6)	2.8 (1–6)
Days in milk	284 (216–431)	291 (228–425)
Reproduction status	All pregnant	All pregnant
Days until parturition	121 (88–155)	118 (73–177)
Total leukocyte count (TLC) (cells/μL)	1.49 × 10^4^ (8.48 × 10^3^–2.69 × 10^4^)	8.63 × 10^3^ (5.30 × 10^3^–1.78 × 10^4^)

*Data represent the mean and (range) on the day of sample collection*.

### Whole Blood, Plasma, and B-Cell Isolation

Whole blood and plasma samples were collected as previously described (8). Briefly, whole blood for BLV PVL quantification and (TLCs) was collected by coccygeal venipuncture into Vacutainer tubes containing the anticoagulant EDTA (Becton Dickinson). Freshly isolated whole blood was used to determine (TLCs). Aliquots of whole blood were stored at −20°C for PVL quantification.

Whole blood was also collected by coccygeal venipuncture into Vacutainer tubes containing the anticoagulant ACD (Becton Dickinson) for plasma and peripheral blood leukocyte (PBL) isolation. Briefly, plasma aliquots with 0.1% sodium azide were stored at −20°C prior to ELISA analysis. PBLs were isolated using red blood cell lysis. Briefly, Vacutainer tubes were centrifuged at 2,200 rpm for 20 min at room temperature and buffy coats were transferred to 50 mL conical tubes. Red blood cell lysis solution (154 mM ammonium chloride, 10 mM potassium bicarbonate, and 97 µM tetrasodium EDTA in deionized water) was added at a ratio of 2:1 to the buffy coats and samples were inverted for 5 min. PBLs were then centrifuged at 1,500 rpm for 5 min at 4°C and washed with 25 mL of 1× phosphate-buffered saline (PBS). PBLs were counted with a hemocytometer.

6 × 10^7^ PBLs were labeled for 30 min at 4°C with 2.5 µg primary anti-bovine IgG2b antibody targeting surface IgM (SIgM) (clone PIG45A2; Washington State University) in 800 µL staining buffer (PBS with 2% heat-inactivated horse serum, 10% ACD, and 0.09% sodium azide). Cells were washed with 3 mL wash buffer (PBS with 10% ACD and 0.09% sodium azide), and centrifuged at 150 × *g* for 5 min at 4°C. Cells were then labeled for 30 min at 4°C with 1 µg secondary anti-mouse IgG2b antibody conjugated to Alexa Fluor 488 (Life Technologies). Cells were washed with 3 mL wash buffer and centrifuged at 150 × *g* for 5 min at 4°C. Cells were suspended in 2 mL 1× Hank’s balanced salt solution (HBSS) and stored at 4°C. Live cells were first selected based on forward and side-scatter gating, and were then positively sorted based on fluorescence using an Influx Cell Sorter (Becton Dickinson). 1.5 × 10^6^ SIgM+ lymphocytes were collected for RNA extraction and qRT-PCR analysis. Data from B-cell sorting were also used to calculate the mean relative percent of SIgM+ B cells.

### TLC Quantification

The TLC was determined using a Z1 Coulter Particle Counter (Beckman Coulter) according to instrument specifications. Briefly, 40 µL of whole blood was diluted in 20 mL 1× PBS and 6 drops of Zap-Oglobin II (Beckman Coulter) to lyse red blood cells. Samples were left at room temperature between 2 and 30 min before running on the particle counter. Each sample was run in triplicate and the average TLC was recorded as cells/μL.

### Total IgM Quantification

Total plasma IgM was quantified using the bovine IgM ELISA quantitation set (Bethyl Laboratories) following the recommended protocol. Briefly, flat-bottomed ELISA plates (Thermo Fisher Scientific) were incubated with 100 µL coating antibody diluted in coating buffer for 1 h at room temperature. Wells were washed 5× with 200 µL wash buffer, and then 200 µL wash buffer was incubated for 30 min at room temperature to block the plate. Wells were washed 5× with 200 µL wash buffer, and 100 µL plasma was diluted in the ratio of 1:10,000 in wash buffer and was incubated for 1 h at room temperature. Wells were washed 5× with 200 µL wash buffer and 100 µL anti-IgM-HRP diluted in the ratio of 1:100,000 in wash buffer and was added to all wells and incubated for 1 h at room temperature. Wells were washed 5× with 200 µL wash buffer, and 100 µL TMB substrate (Thermo Fisher Scientific) was added to all wells and incubated for 15 min at room temperature in the dark. 100 µL of stop solution was added to all wells. The optical density was measured at 450 nm using a SpectraMax M5 microplate reader and total IgM was quantified using a standard curve according to kit protocol. All samples were run in duplicate and plates included blank controls.

### BLV PVL Quantification

DNA was extracted from whole blood using the DNeasy blood and tissue kit (Qiagen) using a modified kit protocol (9). DNA was quantified using a NanoDrop ND-1000 spectrophotometer (Thermo Fisher Scientific) and purity was assessed using A260/280 ratios. qPCR was performed using 60 ng DNA and Power SYBR Master Mix (Applied Biosystems) in 50 µL reaction volumes run in triplicate on a 7500 Fast Real-Time PCR system (Applied Biosystems).

Primers targeting the BLV provirus (BLV; Table 2) (27) or β-actin (ACTB; Table 2) were used to relatively quantify the amount of BLV provirus in BLV+ cows. BLV was normalized to *ACTB* and the abundance of provirus was relatively quantified using 2^−ΔCt^ (28). BLV primers amplified targets at significantly higher expression in BLV+ cows (Figure S1A in Supplementary Material).

**Table 2:** Primer sequences for qPCR and qRT-PCR.

Gene target	Forward primer (5′–3′)	Reverse primer (5′–3′)
BLV (gDNA)	ACT TTC AGA CCC CCT TGA CTG ACA	AAA CCT CTG CCC TGG TGA TTA AGG
ACTB (gDNA)	TCC CTG GAG AAG AGC TAC GA	GGC AGA CTT AGC CTC CAG TG
IGJ (cDNA)	TGA CCC CGG ATT CCT GCT AT	GAT AAG CAG TTG TGC AGC CAG
PRDM1 (cDNA)	AAA CGT GTG GGT ACG ACC TT	CTT CAG TCC CCT CTG CCA AC
PAX5 (cDNA)	AAA ATT ACC CGA CTC CTC GGA C	GTG GCC GTC CAT TCA CAA AAA
BCL6 (cDNA)	AGG AAA CCT CTC ATT TTA GAG TGC	CGG CGA GGC CAT TTT TCT TC
TAX (cDNA)	GGA GCT ACA CCA TTC ACC CC	TCA GAG CCC TTG GGT GTT TC
PPIA (cDNA)	GCA AGC ACG TGG TAC TTT GG	TTG CTG GTC TTG CCA TTC CT
HPRT (cDNA)	TGC ACT ACG AGC CTA AAG AC	TCC AGT CAA TAG TGG TGT GGT
ACTB (cDNA)	TGG AAC GGT GAA GGT GAC AG	CAA TCA AGT CCT CGG CCA CA

*All primer pairs were used at a final concentration of 300 nM per reaction except for PAX5, in which both primers were used at a final concentration of 100 nM per reaction*.

### Cellular Gene Expression Quantification

Total RNA was extracted from 1.5 × 10^6^ SIgM+ B cells using the miRNeasy Mini Kit (Qiagen) according to the manufacturer’s instructions using the on-column DNase digestion protocol. Extracted RNA was quantified using a NanoDrop-1000 and purity was assessed using A260/280 ratios. 71.4 ng RNA was reverse transcribed using the high capacity cDNA reverse transcription kit with random primers (Applied Biosystems). qRT-PCR was performed using 3 µL of cDNA diluted 1:10 and Power SYBR Green PCR Master Mix (Applied Biosystems) in 50 µL reaction volumes run in triplicate on a 7500 Real-Time PCR system (Applied Biosystems).

*IGJ, PAX5, BLIMP1, BCL6, TAX, PPIA* (29), *HPRT*, and *ACTB* were assayed for each cow (Table 2). NormFinder software (30) was used to determine the most stably expressed internal control genes (*PPIA, HPRT*, and β-actin). Subsequently, *PPIA* alone was used to relatively quantify *IGJ, PAX5, BLIMP1, BCL6*, and *TAX* expression. Expression in both BLV+ and BLV− cows was assessed using 2^−ΔCt^ (28). *TAX* primers amplified targets at significantly higher expression in BLV+ cows (Figure S1B in Supplementary Material).

### BLV miRNA Expression Quantification

10 ng of extracted RNA from SIgM+ B cells was reversed transcribed using the high capacity cDNA reverse transcription kit (Applied Biosystems). BLV miRNA B4-3p, B5-5p, B3-3p, B1-3p, and B2-5p expression was measured using stem-loop reverse transcription primers (Thermo Fisher Scientific) and Taqman microRNA assays (Thermo Fisher Scientific). U6 was used as an endogenous control and *C. elegans* miR-39 (Cel-39) (Norgen Biotek Corp.) was added to the RT reaction as a spike-in control. Samples were assayed in triplicate on a 7500 Real-Time PCR system (Applied Biosystems). BLV miRNA expression was relatively quantified using 2^−ΔΔCt^ (28). BLV miRNA and U6 expression were normalized to Cel-39 and then BLV miRNA expression was normalized to U6 expression (31). BLV miRNA primers amplified targets at significantly higher expression in BLV+ cows (Figures S1C,D,F,G in Supplementary Material) or failed to amplify targets in BLV− cows (Figure S1E in Supplementary Material).

### Statistical Analysis

Normality was assessed using the Shapiro–Wilk test. Outliers were detected using Grubbs test and removed from analysis. Significance between BLV+ and BLV− cows was assessed using a Student’s unpaired *t*-test. Correlation between parameters in BLV+ cows was assessed by calculating the Pearson correlation coefficients. Data were analyzed using Prism 7.0b (GraphPad Software) and significance was determined as *p* < 0.05.

### Plasma IgM Quantification from Previous Studies

In two previous studies, adult Holstein BLV+ and BLV− cows were exposed to a routine vaccination with Bovi-Shield GOLD^®^ FP^®^ 5 L5 HB (8) or to a novel antigen (9). Prior to cattle exposure, whole blood was collected by coccygeal venipuncture and plasma aliquots containing 0.1% sodium azide were stored at −80°C. Total IgM quantification and statistical analysis were conducted as described here.

## Results

### BLV+ Cows Demonstrate Reduced Antigen-Specific and Total Plasma IgM

**Figure 1:**
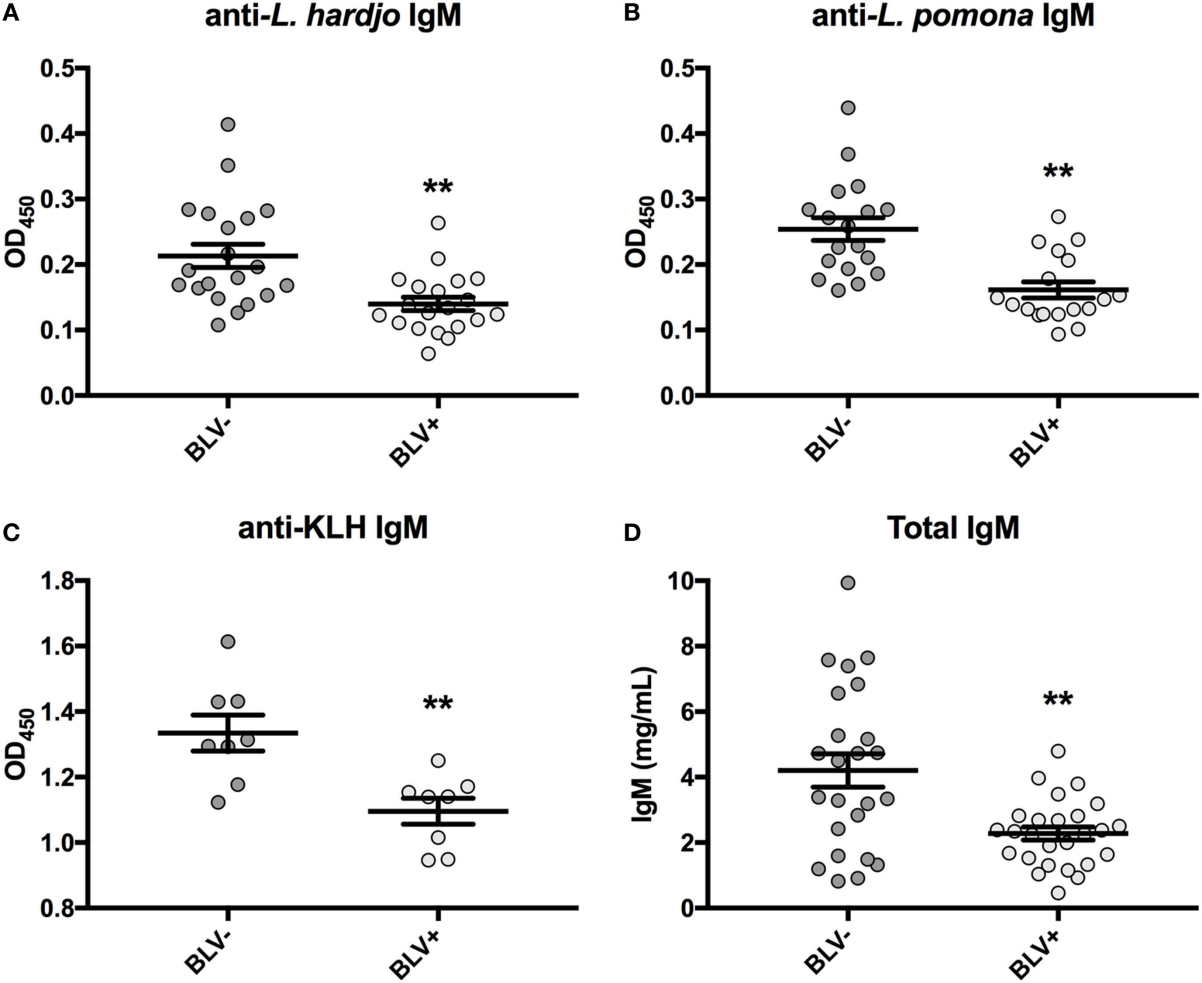
IgM in plasma from cattle enrolled in previous studies. All plasma samples were collected on day 0 prior to vaccination or antigen exposure. **(A)** Anti-*L. hardjo* IgM (BLV+ *n* = 20, BLV- *n* = 20), **(B)** anti-*L. pomona* IgM (BLV+ *n* = 18, BLV− *n* = 18), **(C)** anti-KLH IgM (BLV+ *n* = 8, BLV− *n* = 8), and **(D)** total plasma IgM (BLV+ *n* = 26, BLV− *n* = 24). Bars indicate the mean ± SEM.

Previous research had found reduced antigen-specific plasma IgM in BLV+ cows over a period of 1 or 3 months after antigen exposure (8, 9). Upon reanalysis of data collected on day 0, only reduced antigen-specific IgM was even observed on day 0 prior to vaccination or antigen exposure (Figures 1A–C). If lower observed IgM levels were the result of reduced *IGJ* expression in BLV+ cows, then the total plasma IgM concentration should also be reduced in those BLV+ cattle. Indeed, total IgM concentrations in plasma collected on day 0 from cattle enrolled in the previous time course studies (8, 9) were significantly reduced in BLV+ cows relative to their uninfected herdmates (*p* = 0.0007) (Figure 1D).

### Higher Levels of BLV *TAX* and miRNA Expression Are Associated with Increasing PVL

**Figure 2:**
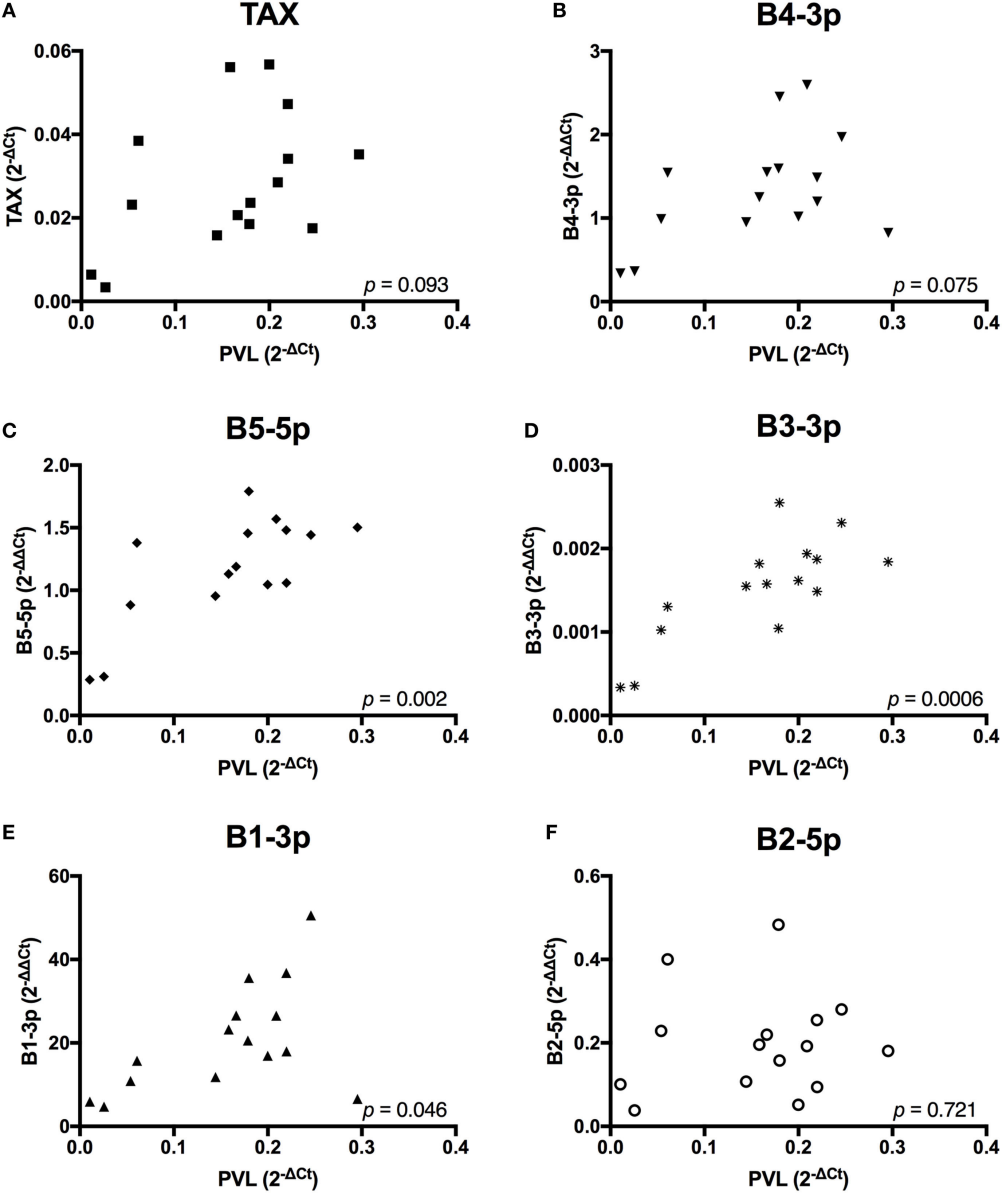
Proviral load (PVL) correlations with BLV *TAX* or BLV microRNAs (miRNAs). PVL in BLV+ cows correlated with **(A)** *TAX*, **(B)** B4-3p, **(C)** B5-5p, **(D)** B3-3p, **(E)** B1-3p, and **(F)** B2-5p. *n* = 15.

Given the observed lower plasma IgM in BLV+ cows, we wanted to investigate whether BLV gene expression or miRNA expression correlated with any changes in gene expression or abnormal phenotypes in BLV+ cows. To do so, it was critical to establish how different measurements of viral activity correlated with each other in order to interpret any significant associations that were detected between viral expression and host phenotypes. PVL was significantly, positively correlated to miRNAs B5-5p, B3-3p, and B1-3p. PVL also exhibited a trending positive correlation with both *TAX* and miRNA B4-3p (Figure 2).

**Figure 3:**
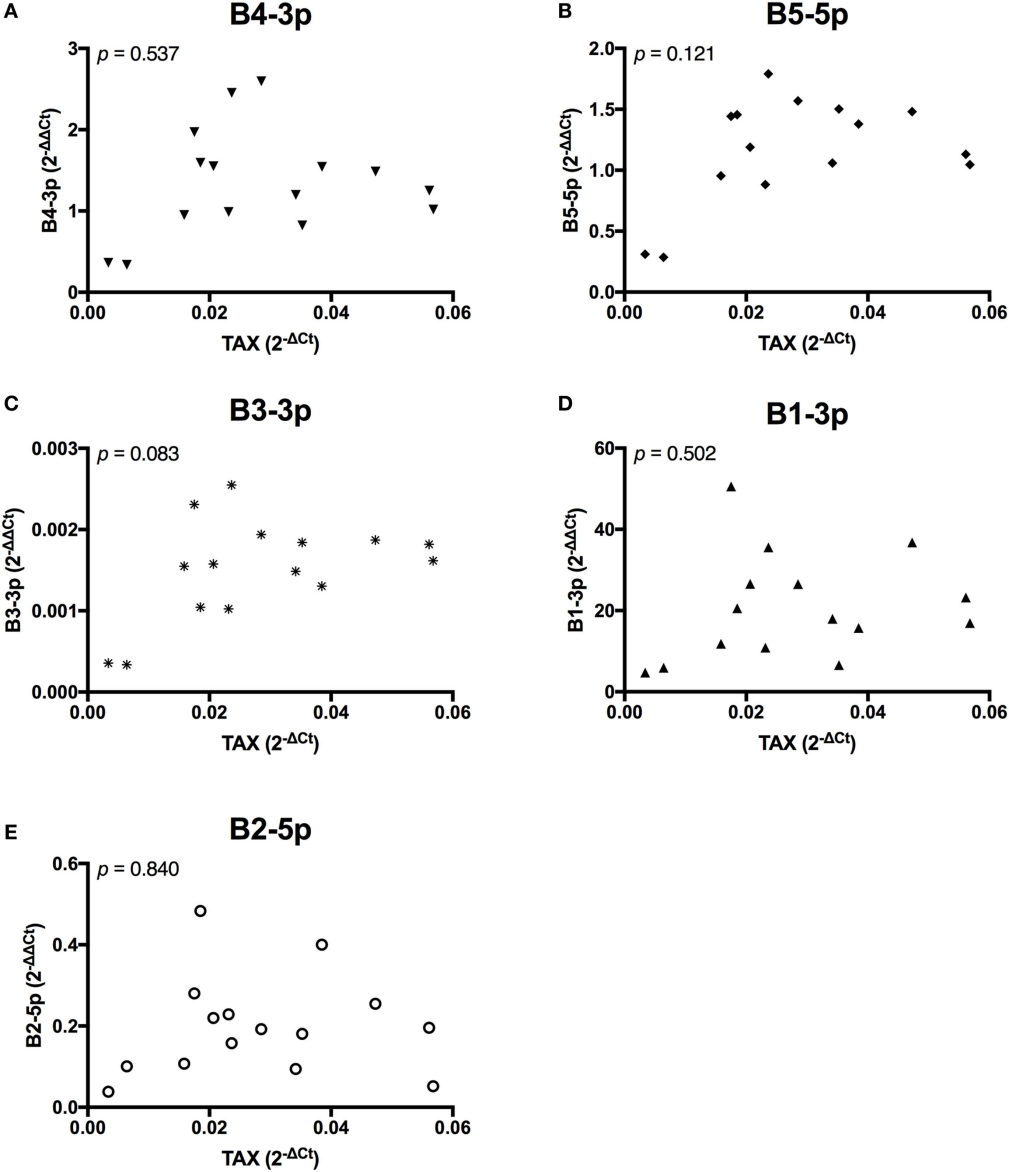
*TAX* correlations with BLV MicroRNAs. *TAX* in BLV+ cows correlated with **(A)** B4-3p, **(B)** B5-5p, **(C)** B3-3p, **(D)** B1-3p, and **(E)** B2-5p. *n* = 15.

**Figure 4:**
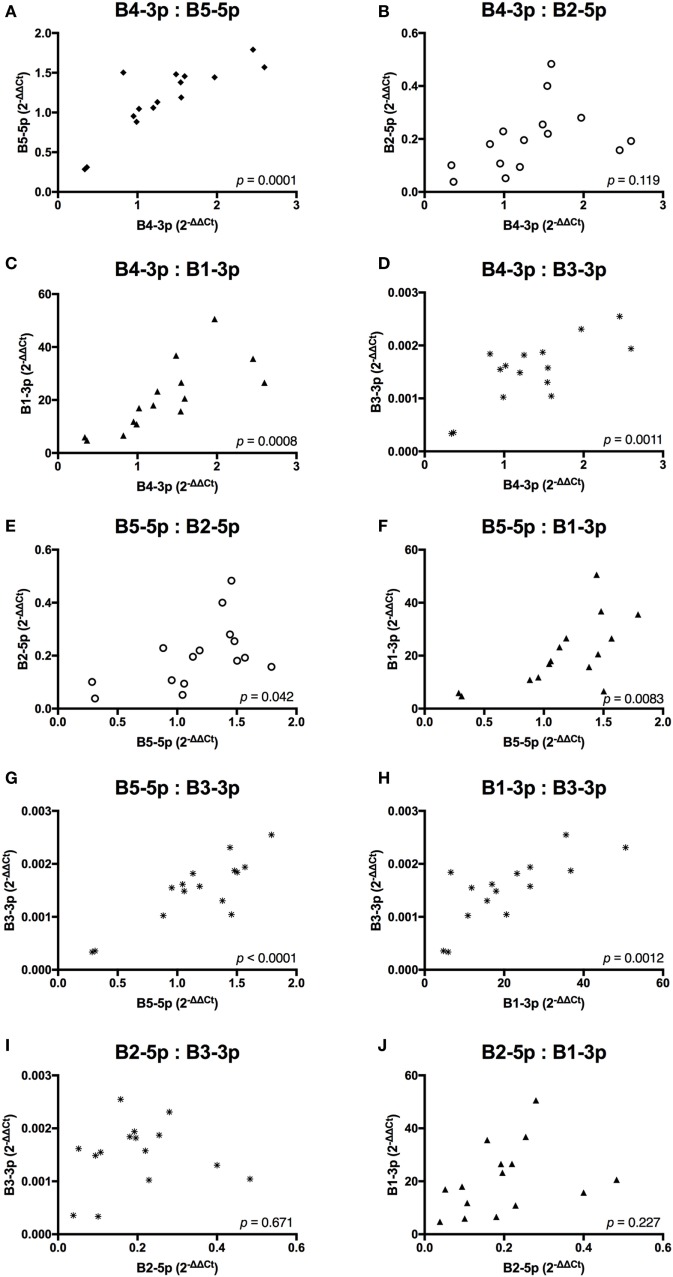
BLV MicroRNAs correlations. **(A)** B4-3p: B5-5p, **(B)** B4-3p: B2-5p, **(C)** B4-3p: B1-3p, **(D)** B4-3p: B3-3p, **(E)** B5-5p: B2-5p, **(F)** B5-5p: B1-3p, **(G)** B5-5p: B3-3p, **(H)** B1-3p: B3-3p, **(I)** B2-5p: B3-3p, and **(J)** B2-5p: B1-3p. *n* = 15.

In contrast, *TAX* expression only exhibited a trending positive correlation with the expression of one miRNA, B3-3p (Figure 3). When comparing BLV miRNA expression between individual miRNAs, most measured miRNAs significantly, positively correlated with each other. However, B2-5p expression was only positively associated with B5-5p (Figure 4).

Taken together, these data suggest that expression of both BLV *TAX* and BLV miRNA is higher in animals with increasing PVL. However, BLV *TAX* and BLV miRNA expression levels do not appear to be related to each other. In addition, four BLV miRNAs (B4-3p, B5-5p, B3-3p, and B1-3p) show closely correlated expression levels, while one BLV miRNA (B2-5p) does not.

### B Cells from BLV+ Cows Demonstrate Reduced *IGJ* Expression

**Figure 5:**
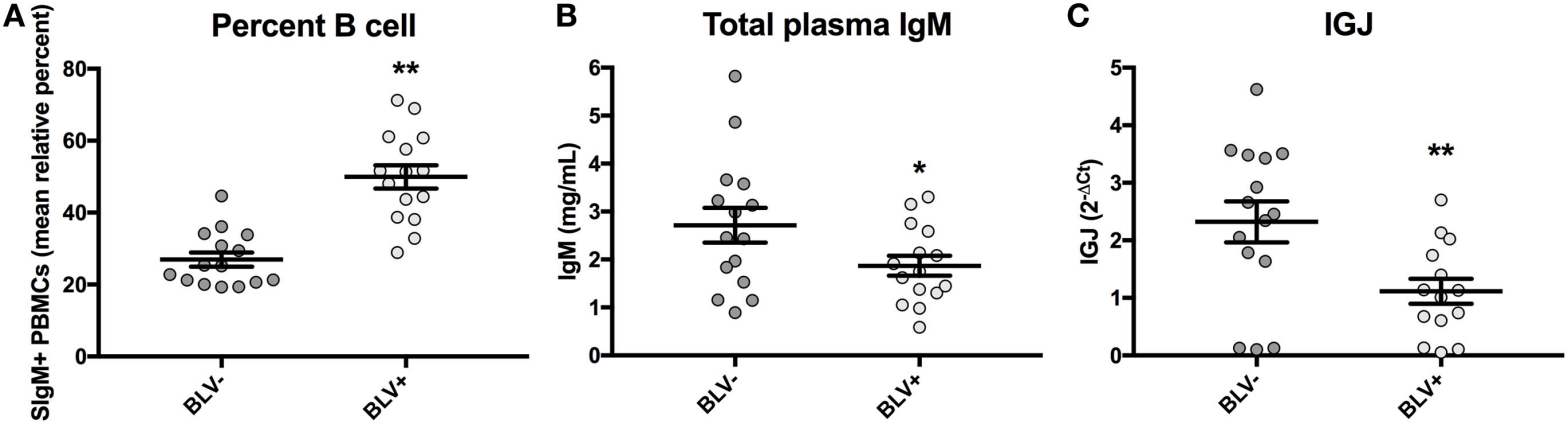
Percent B-cell prevalence, total plasma IgM, and *IGJ* expression in BLV+ and BLV− cows. **(A)** Percent B-cell prevalence (BLV+ *n* = 15, BLV− *n* = 15), **(B)** total plasma IgM (BLV+ *n* = 15, BLV− *n* = 15), and **(C)** *IGJ* expression (BLV+ *n* = 14, BLV− *n* = 15). Bars indicate the mean ± SEM.

BLV+ cows in this study were found to exhibit a significant increase in the prevalence of SIgM+ B cells in peripheral blood (*p* < 0.0001) (Figure 5A), as expected. Similarly to the results obtained from a larger sample size, there was a near-significant reduction in total plasma IgM (*p* = 0.053) (Figure 5B). When the relative expression of *IGJ* in SIgM+ B cells from BLV+ and BLV− cows was compared, BLV+ cows demonstrated a significant twofold reduction (*p* = 0.008) (Figure 5C).

**Figure 6:**
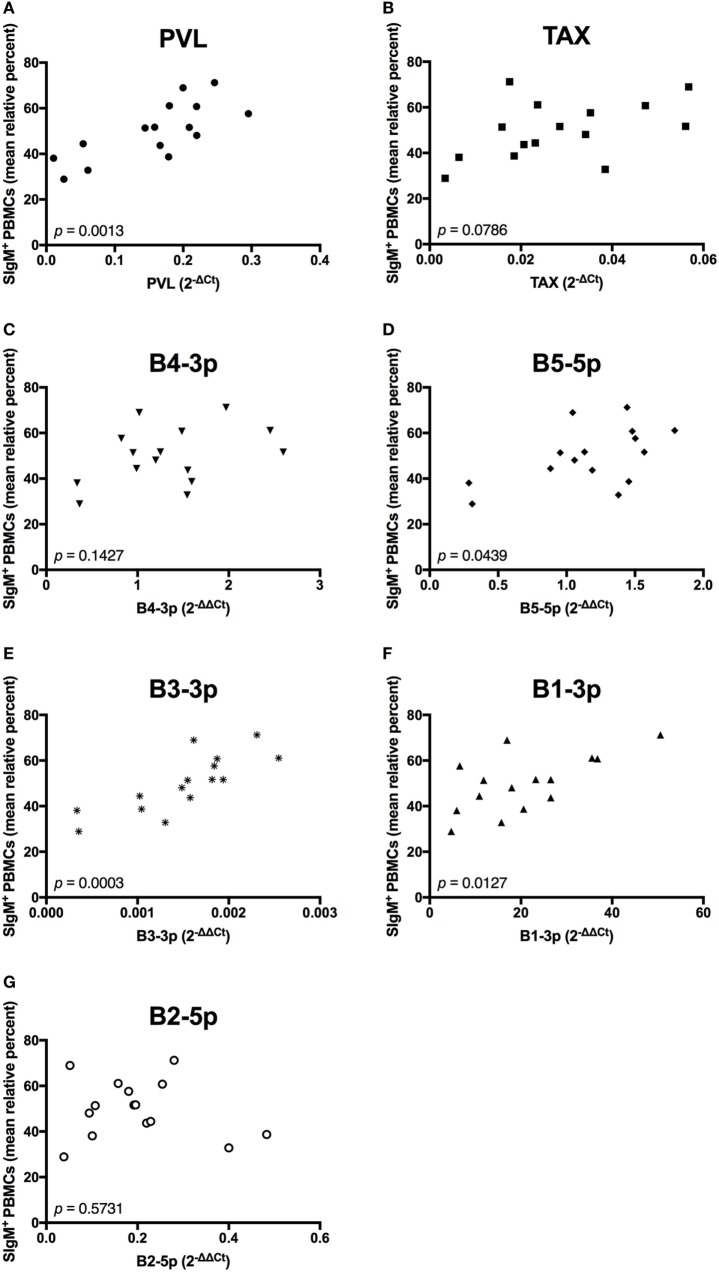
Percent B-cell prevalence and BLV expression in BLV+ cows. The percent B-cell prevalence in BLV+ cows correlated with **(A)** PVL, **(B)** *TAX*, **(C)** B4-3p, **(D)** B5-5p, **(E)** B3-3p, **(F)** B1-3p, and **(G)** B2-5p. *n* = 15.

We next wanted to determine whether BLV PVL or BLV activity correlated to the observed phenotypic abnormalities in BLV+ cows. PVL, B5-5p, B3-3p, and B1-3p expression were all positively correlated to the percentage of SIgM+ B cells. However, only a trending positive association was observed between *TAX* and the percentage of B cells (Figure 6).

**Figure 7:**
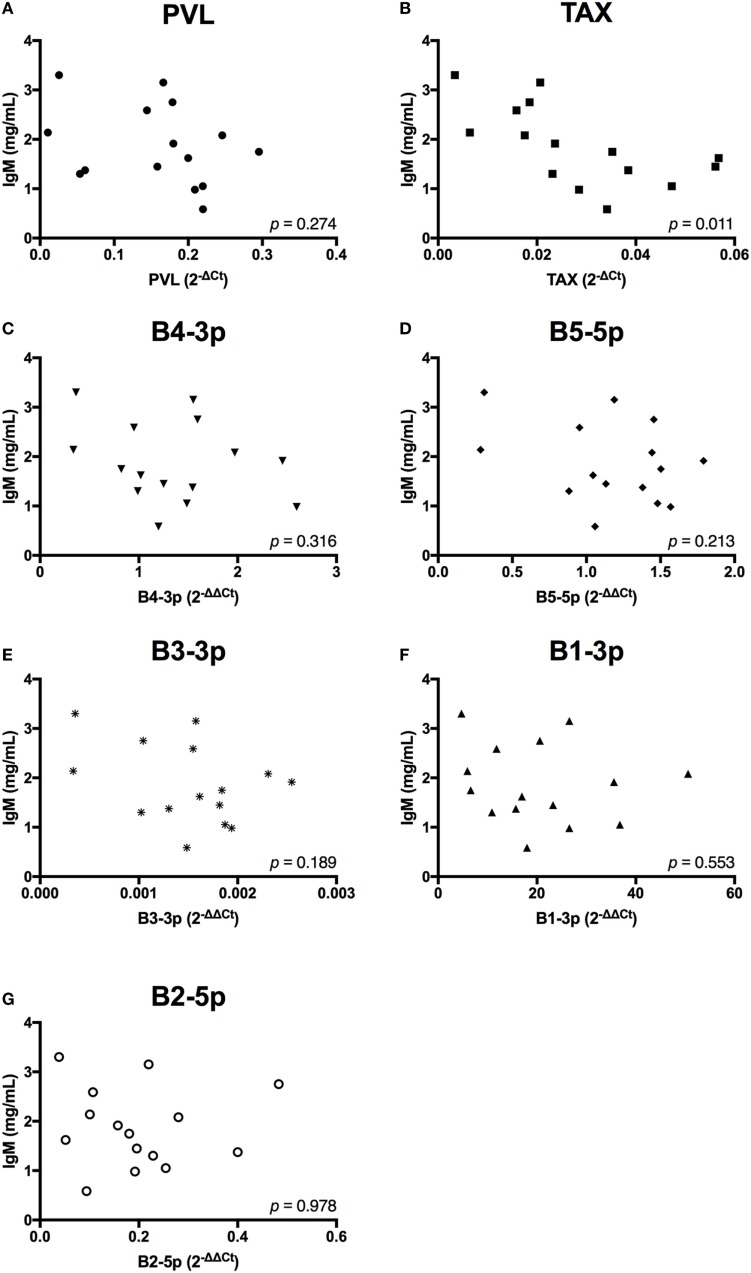
Total plasma IgM and BLV expression in BLV+ cows. The concentration of total plasma IgM in BLV+ cows correlated with **(A)** proviral load (PVL), **(B)** *TAX*, **(C)** B4-3p, **(D)** B5-5p, **(E)** B3-3p, **(F)** B1-3p, and **(G)** B2-5p. *n* = 15.

**Figure 8:**
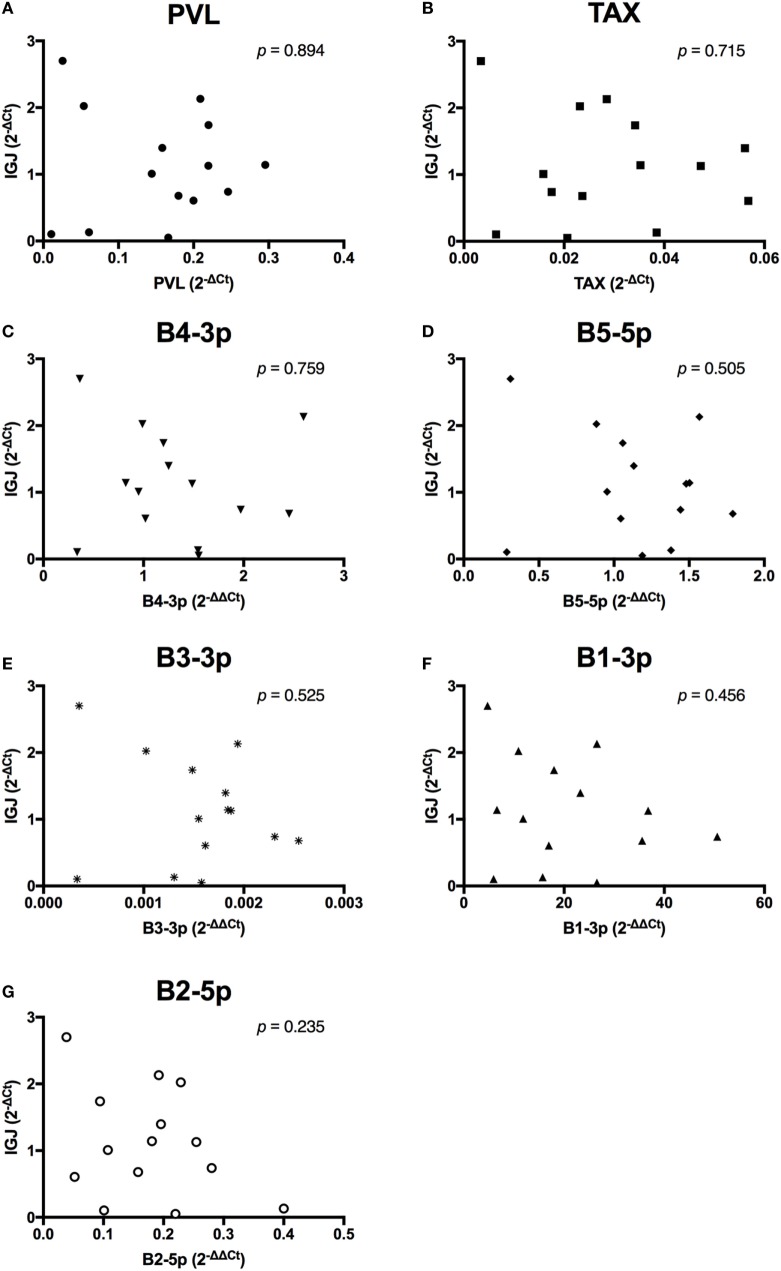
*IGJ* expression and BLV expression in BLV+ cows. The expression of *IGJ* in BLV+ cows correlated with **(A)** proviral load (PVL), **(B)** *TAX*, **(C)** B4-3p, **(D)** B5-5p, **(E)** B3-3p, **(F)** B1-3p, and **(G)** B2-5p. *n* = 14.

In contrast to the observed associations between viral expression and the percentage of B cells, only *TAX* expression exhibited a negative association with total plasma IgM (Figure 7). Finally, when the relationship between viral miRNA expression and *IGJ* expression was examined, no associations were detected, despite the overall reduction in *IGJ* expression in BLV+ cows (Figure 8).

### B Cells from BLV+ Cows Exhibit Decreased *BLIMP1* and *BCL6* Expression

**Figure 9:**
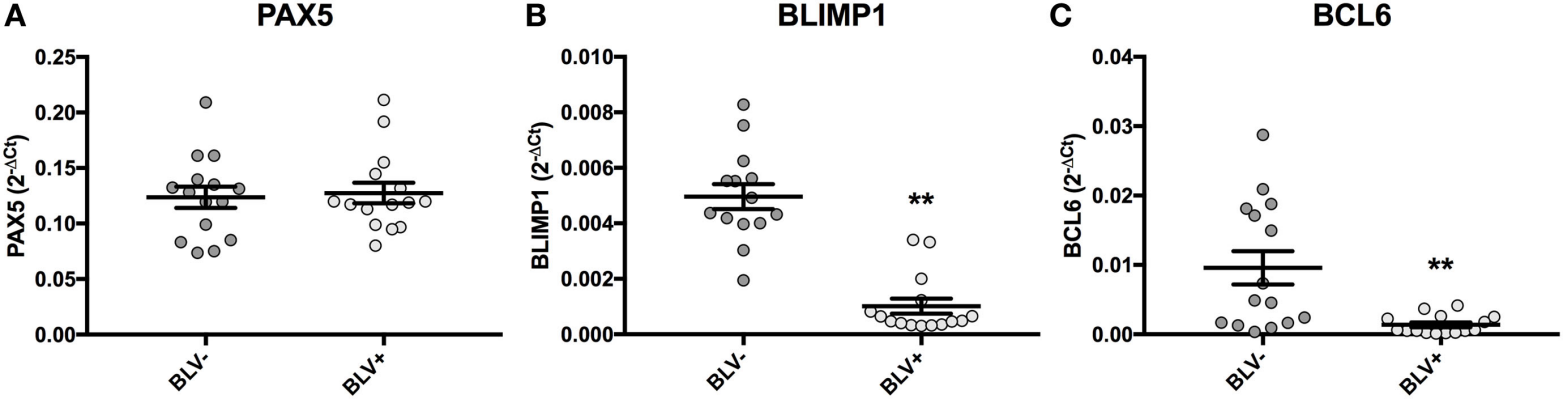
*PAX5, BLIMP1*, and *BCL6* expression in BLV+ and BLV− cows. **(A)** *PAX5* expression (BLV+ *n* = 15, BLV− *n* = 15), **(B)** *BLIMP1* expression (BLV+ *n* = 15, BLV− *n* = 14), and **(C)** *BCL6* expression (BLV+ *n* = 15, BLV− *n* = 15). Bars indicate the mean ± SEM.

Although BLV+ cows were found to express less *IGJ* in SIgM+ B cells, lower *IGJ* expression did not correlate to any measurements of viral gene or miRNA expression. To investigate how BLV expression might indirectly affect *IGJ* expression *in vivo*, transcription factors regulating B-cell differentiation were subsequently analyzed. While BLV+ cows demonstrated no difference in *PAX5* expression (Figure 9A), they did show a significant fivefold reduction in *BLIMP1* expression (*p* < 0.0001) (Figure 9B), and a significant sevenfold reduction in *BCL6* expression (*p* = 0.002) (Figure 9C).

**Figure 10:**
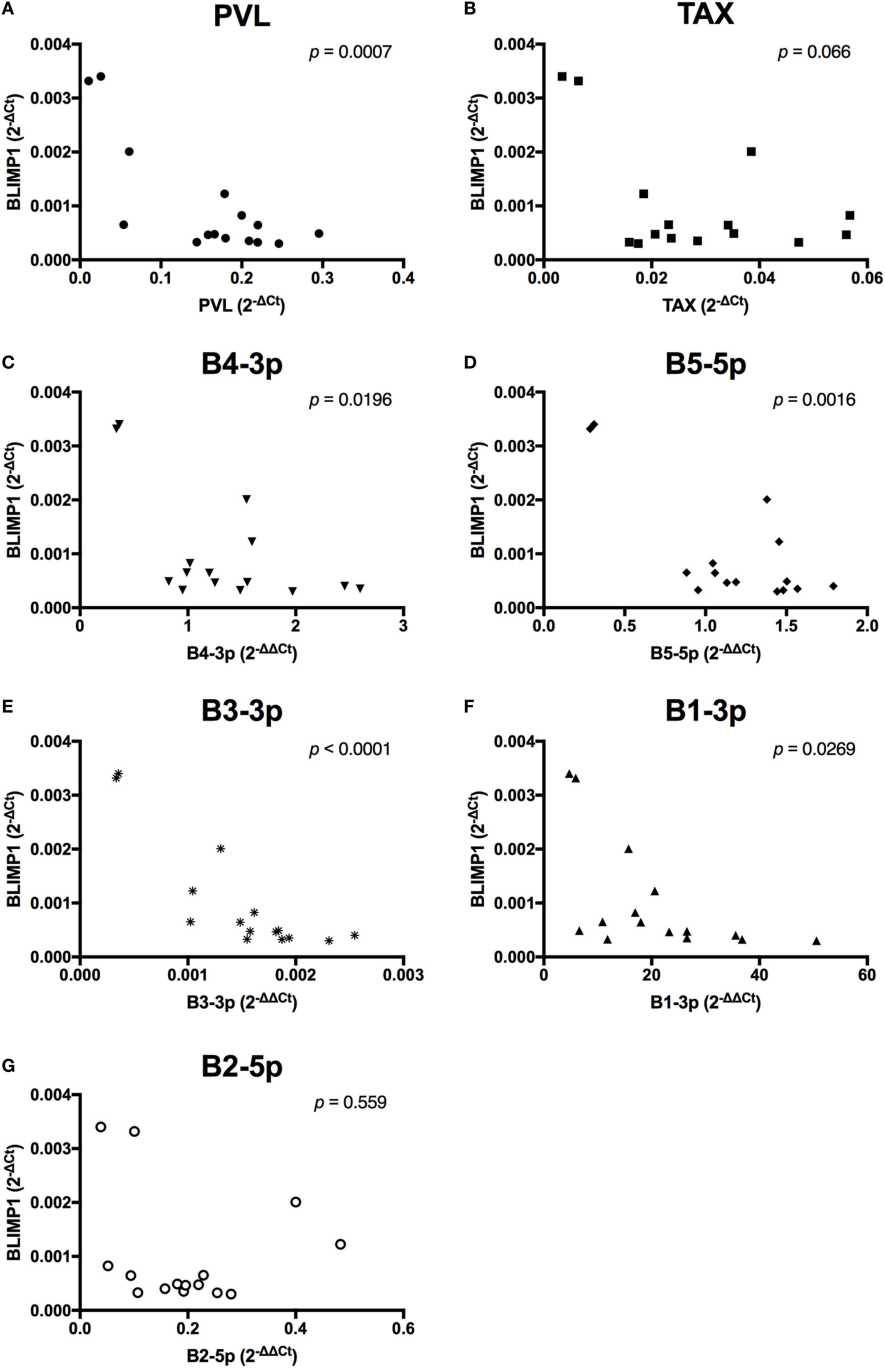
*BLIMP1* expression and BLV expression in BLV+ cows. The expression of *BLIMP1* in BLV+ cows was correlated with **(A)** proviral load (PVL), **(B)** *TAX*, **(C)** B4-3p, **(D)** B5-5p, **(E)** B3-3p, **(F)** B1-3p, and **(G)** B2-5p. *n* = 15.

When viral expression was compared with *BLIMP1* expression in BLV+ cows, the PVL (Figure 10A), expression of B4-3p, B5-5p, B3-3p, and B1-3p were all negatively associated with *BLIMP1* expression. *TAX* expression also demonstrated a trending negative association with *BLIMP1* expression (Figure 10).

**Figure 11:**
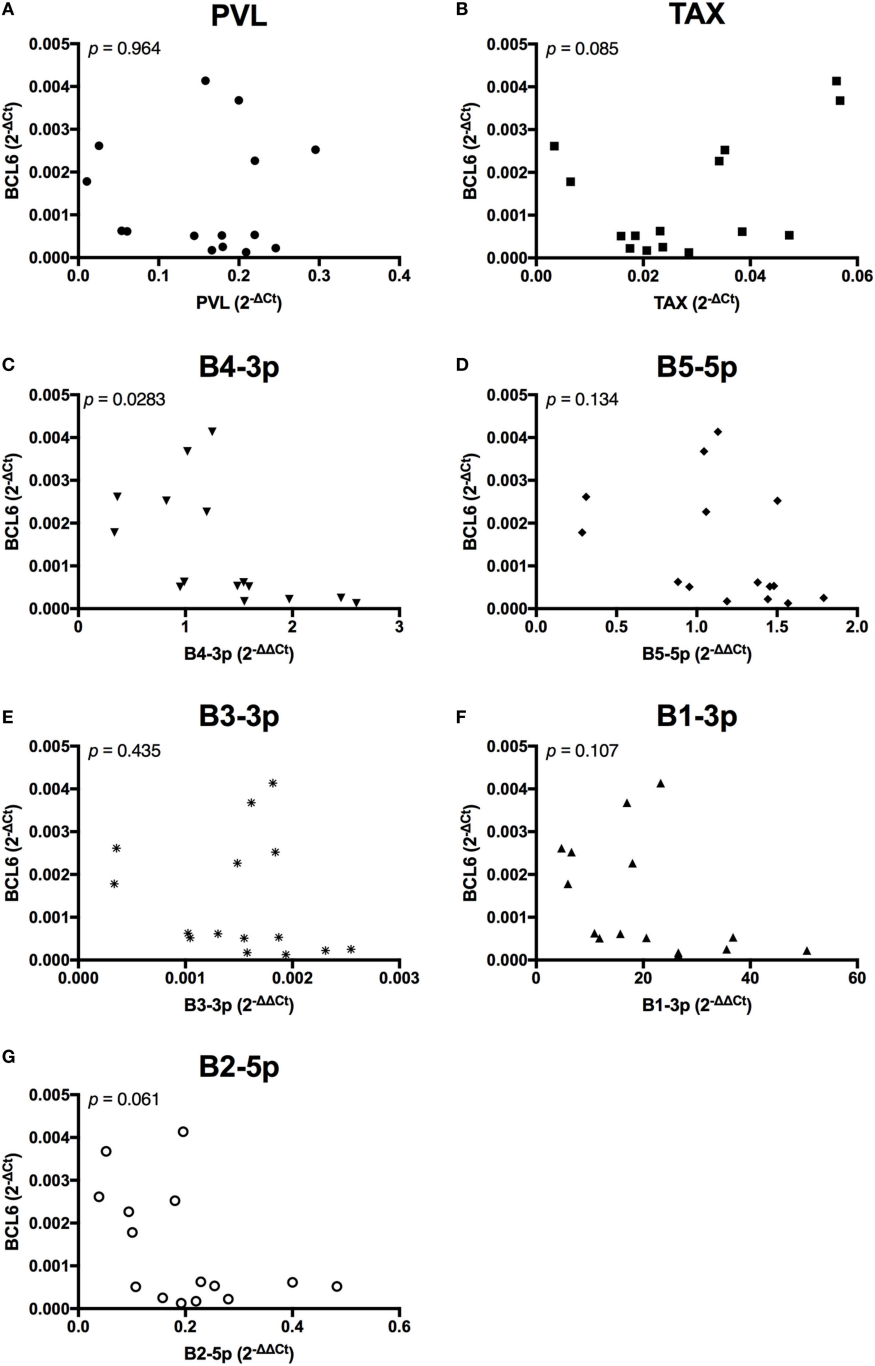
*BCL6* expression and BLV expression in BLV+ cows. The expression of *BCL6* in BLV+ cows correlated with **(A)** proviral load (PVL), **(B)** *TAX*, **(C)** B4-3p, **(D)** B5-5p, **(E)** B3-3p, **(F)** B1-3p, and **(G)** B2-5p. *n* = 15.

Unlike the associations observed between viral expression and *BLIMP1* expression, *BCL6* expression was only negatively associated with B4-3p, although a trending negative association was also observed with B2-5p expression. Interestingly, a trending positive association was also observed between *TAX* and *BCL6* (Figure 11).

## Discussion

In this study, we investigated the impact of BLV infection on the concentration of total IgM in naturally infected dairy cows, as well as potential viral factors driving reduced IgM. IgM production was investigated, because (1) our research had previously found reduced antigen-specific IgM in BLV+ cows after vaccination (8) and after primary antigenic exposure (9) and (2) IgM is functionally important for protection against infectious agents. Natural IgM is produced in the absence of antigenic stimulation and recognizes pathogen-associated molecular patterns, including carbohydrates, phospholipids, and nucleic acids (32) and is an important component of innate immunity. In addition, IgM is also important during the initial adaptive immune response through its early production and high avidity. In cattle, IgM is especially effective at activating complement against extracellular bacteria and has been found to delay or prevent the onset of experimentally induced *E. coli* septicemia in neonatal calves (33, 34).

Bovine leukemia virus has two distinct known mechanisms that could ultimately affect IgM production includes: (1) the transcription and production of viral proteins, including *TAX* (12), and (2) the transcription of virally encoded miRNAs (19). *TAX* is the transactivator of BLV transcription and a candidate as a driver of BLV-induced oncogenesis (12). However, a puzzling aspect of BLV pathogenesis in cattle without lymphoma is the difficulty in detecting viral transcripts or proteins *in vivo* (35, 36). Evidence suggests that BLV persistence favors transcriptionally inactive provirus (37), which is likely the result of the rapid clearance of infected cells expressing BLV (14). In recent years, BLV miRNAs have emerged as a possible source of observed immune abnormalities in BLV+ cows. BLV miRNAs have been observed *in vivo* in both experimentally infected sheep (16) and calves (19). In fact, comparing the transcriptomes of cells infected with WT BLV to cells infected with a mutant BLV missing the entire miRNA-encoding cassette demonstrated that WT-infected cells specifically exhibited downregulated *IGJ* expression (19). As a result, we investigated whether BLV miRNA expression correlated to decreased expression of genes important for antibody production, which would suggest possible direct targets of BLV miRNA activity and suppression.

Although BLV+ cows exhibited a significant twofold reduction in *IGJ* expression in comparison to BLV− cows, *IGJ* expression was not correlated to any measure of viral activity, suggesting that *IGJ* is not a direct target of BLV miRNAs. When the expression of transcription factors *PAX5, BLIMP1*, and *BCL6* were examined, both *BLIMP1* and *BCL6* were found to be significantly downregulated in BLV+ cows. *BLIMP1* expression was negatively correlated with the expression of four out of five tested BLV miRNAs, suggesting that *BLIMP1* may be the direct target of one or more BLV miRNAs. In addition, *BLIMP1* also exhibited a trending negative association with *TAX* expression, although it is unclear how *TAX* would interfere with *BLIMP1* expression. In fact, it is possible that elevated *BLIMP1* expression could suppress *TAX* expression by interfering with BLV transcription driven by endogenous factors. Bovine IRF1 and IRF2 (38) and Spi-B (39) transcription factors can bind to the BLV promoter and induce transcription. *BLIMP1* can antagonistically bind to both IRF1 and IRF2 target sequences (40) and directly repress the expression of Spi-B (41).

In contrast to the results observed with *BLIMP1*, only one BLV miRNA exhibited a significant negative correlation with *BCL6* expression, although a second BLV miRNA did exhibit a trending negative correlation. What was most intriguing was a trending positive association between *TAX* and *BCL6*. This was the only measurement where *TAX* and BLV miRNAs demonstrated opposite associations with the target gene. In addition, elevated *BCL6* expression is associated with B-cell lymphoma development in mice (23) and *TAX* is associated with the accumulation of DNA damage and can induce transformation *in vitro* (12). The data suggest that *BCL6* may be directly repressed by one or more BLV miRNAs, while *TAX* expression might actually promote *BCL6* expression.

Overall, this study has illustrated that expression of both BLV miRNAs and BLV *TAX* can be detected in SIgM+ B cells isolated from the periphery of naturally infected dairy cows. Moreover, the negative association between BLV miRNAs or BLV *TAX* and *BLIMP1* or *BCL6* suggests that BLV activity might suppress genes that are essential for either plasma cell differentiation or germinal center formation. Importantly, our results do not suggest that BLV interferes with IgM production specifically, but more likely interferes with overall antibody production. Although we have not previously observed a consistent decrease in plasma IgG (8, 9), it is possible that IgM levels are more obviously affected because of the presence of natural IgM, which is enhanced by *BLIMP1* (42), or that T cell signaling during class switching lessens the impact of BLV infection on B-cell functions. Interestingly, reduced BCL6 is associated with impaired germinal center reactions and affinity maturation, even while maintaining a normal concentration of IgG (25), but it remains to be seen if class-switched antibodies in BLV+ cows have reduced affinities. What is clear is that B cells from BLV+ cows exhibit reduced expression of transcriptional regulators of antibody production and reduced levels of plasma IgM, supporting a possible mechanism through which BLV infection interferes with host immune function.

## Ethics Statement

All animal use protocols were reviewed and approved by Michigan State University Institutional Animal Use and Care Committee (AUF# 04/16-061-00) and written permission was obtained from the commercial dairy herd owner.

## Author Contributions

MF led the study and was involved in the study conception, design, execution, analysis, and interpretation of data. MF wrote the manuscript. CD contributed to study conception, design, execution, and interpretation of data, and manuscript editing. AG contributed to the study execution and manuscript editing. PC contributed to the study conception, design, and interpretation, and manuscript editing.

## Conflict of Interest Statement

Author CD is employed by NorthStar Cooperative. All other authors declare no competing interests. NorthStar Cooperative shared the costs of the ABI 7500 Fast Real-Time PCR system repair. NorthStar Cooperative also paid for reagents and personnel to perform microRNA assays contained within the manuscript.

## Supplementary Material

Figure S1 Relative expression of BLV targets in BLV+ and BLV− cows. **(A)** PVL (BLV+ *n* = 15, BLV− *n* = 3), **(B)** *TAX* (BLV+ *n* = 15, BLV− *n* = 15), **(C)** B4-3p (BLV+ *n* = 15, BLV− *n* = 15), **(D)** B5-5p (BLV+ *n* = 15, BLV− *n* = 15), **(E)** B3-3p (BLV+ *n* = 15, BLV− *n* = 15), **(F)** B1-3p (BLV+ *n* = 15, BLV− *n* = 15), and **(G)** B2-5p (BLV+ *n* = 15, BLV− *n* = 15). Bars indicate the mean ± SEM.
