# Supplementary figures and images for: MicroRNAs Encoded by Bovine Leukemia Virus (BLV) Are Associated with Reduced Expression of B Cell Transcriptional Regulators in Dairy Cattle Naturally Infected with BLV
Source: Front Vet Sci. 2018 Jan 15;4:245. doi: 10.3389/fvets.2017.00245 (PMC5775267; doi:10.3389/fvets.2017.00245)

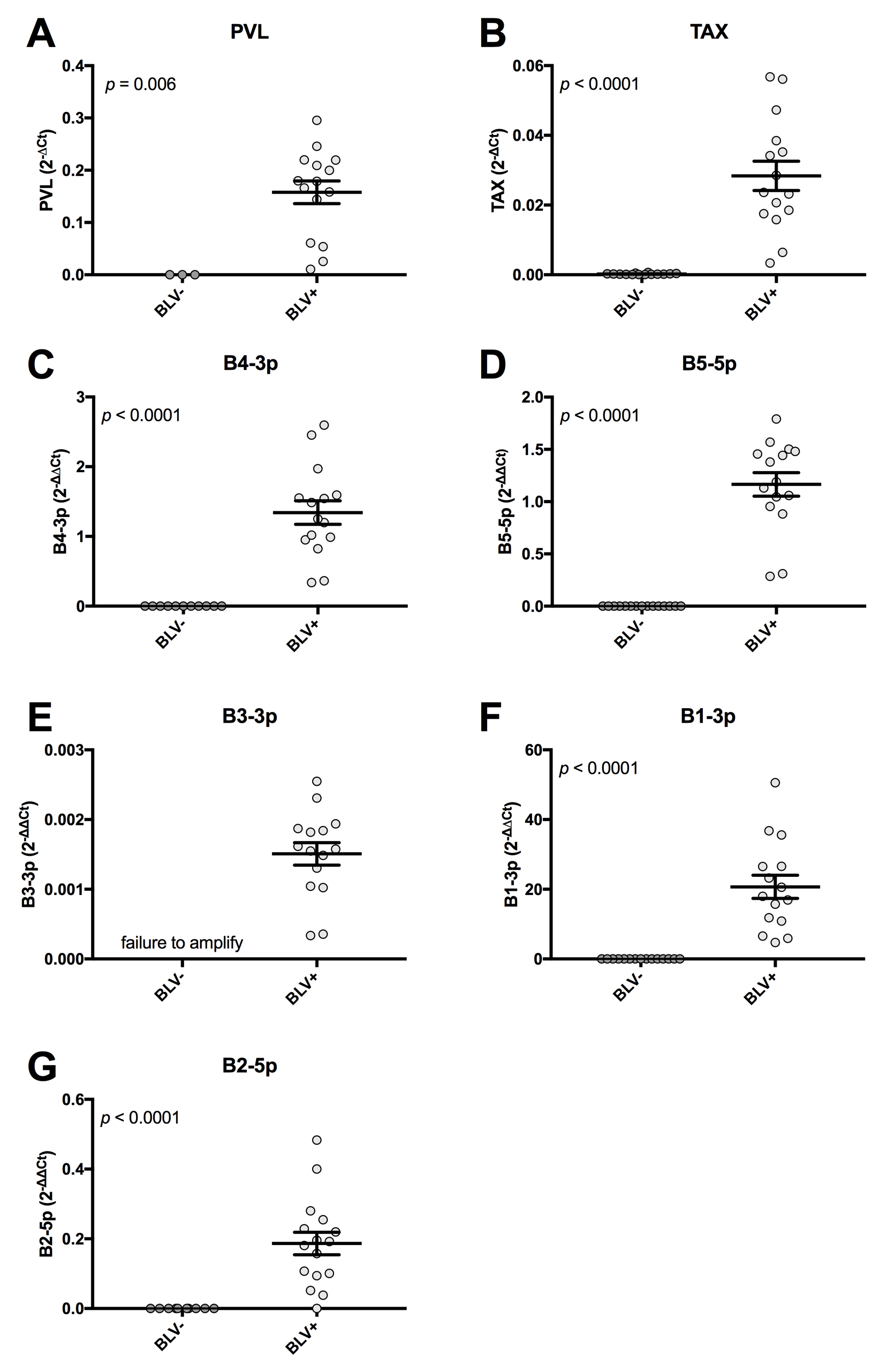

Supplement: Figure S1 — Relative expression of BLV targets in BLV+ and BLV− cows. (A) PVL (BLV+ n = 15, BLV− n = 3), (B) TAX (BLV+ n = 15, BLV− n = 15), (C) B4-3p (BLV+ n = 15, BLV− n = 15), (D) B5-5p (BLV+ n = 15, BLV− n = 15), (E) B3-3p (BLV+ n = 15, BLV− n = 15), (F) B1-3p (BLV+ n = 15, BLV− n = 15), and (G) B2-5p (BLV+ n = 15, BLV− n = 15). Bars indicate the mean ± SEM. [file Image_1.TIFF]
